# Expression of KOC, S100P, mesothelin and MUC1 in pancreatico-biliary adenocarcinomas: development and utility of a potential diagnostic immunohistochemistry panel

**DOI:** 10.1186/1472-6890-14-35

**Published:** 2014-07-23

**Authors:** Asif Ali, Victoria Brown, Simon Denley, Nigel B Jamieson, Jennifer P Morton, Colin Nixon, Janet S Graham, Owen J Sansom, C Ross Carter, Colin J McKay, Fraser R Duthie, Karin A Oien

**Affiliations:** 1 Wolfson Wohl Cancer Research Centre, Institute of Cancer Sciences, College of Medical Veterinary and Life Sciences, University of Glasgow, Garscube Estate, Switchback Road, Bearsden G61 1QH, UK; 2 Pathology Laboratory, Forth Valley Royal Hospital, Stirling Road, Larbert FK5 4WR, UK; 3 West of Scotland Pancreatic Unit and Glasgow Royal Infirmary, Alexandra Parade, Glasgow G31 2ER, UK; 4 Beatson Institute for Cancer Research, Glasgow G61 1BD, UK; 5 Medical Oncology, Beatson West of Scotland Cancer Centre, Glasgow G12 0YN, UK; 6 Department of Pathology, Southern General Hospital, Greater Glasgow & Clyde NHS, Glasgow G51 4TF, UK

**Keywords:** Pancreatic cancer, Biomarkers, Immunohistochemistry, Diagnosis

## Abstract

**Background:**

Pancreatico-biliary adenocarcinomas (PBA) have a poor prognosis. Diagnosis is usually achieved by imaging and/or endoscopy with confirmatory cytology. Cytological interpretation can be difficult especially in the setting of chronic pancreatitis/cholangitis. Immunohistochemistry (IHC) biomarkers could act as an adjunct to cytology to improve the diagnosis. Thus, we performed a meta-analysis and selected KOC, S100P, mesothelin and MUC1 for further validation in PBA resection specimens.

**Methods:**

Tissue microarrays containing tumour and normal cores in a ratio of 3:2, from 99 surgically resected PBA patients, were used for IHC. IHC was performed on an automated platform using antibodies against KOC, S100P, mesothelin and MUC1. Tissue cores were scored for staining intensity and proportion of tissue stained using a Histoscore method (range, 0–300). Sensitivity and specificity for individual biomarkers, as well as biomarker panels, were determined with different cut-offs for positivity and compared by summary receiver operating characteristic (ROC) curve.

**Results:**

The expression of all four biomarkers was high in PBA versus normal ducts, with a mean Histoscore of 150 vs. 0.4 for KOC, 165 vs. 0.3 for S100P, 115 vs. 0.5 for mesothelin and 200 vs. 14 for MUC1 (p < .0001 for all comparisons). Five cut-offs were carefully chosen for sensitivity/specificity analysis. Four of these cut-offs, namely 5%, 10% or 20% positive cells and Histoscore 20 were identified using ROC curve analysis and the fifth cut-off was moderate-strong staining intensity. Using 20% positive cells as a cut-off achieved higher sensitivity/specificity values: KOC 84%/100%; S100P 83%/100%; mesothelin 88%/92%; and MUC1 89%/63%. Analysis of a panel of KOC, S100P and mesothelin achieved 100% sensitivity and 99% specificity if at least 2 biomarkers were positive for 10% cut-off; and 100% sensitivity and specificity for 20% cut-off.

**Conclusion:**

A biomarker panel of KOC, S100P and mesothelin with at least 2 biomarkers positive was found to be an optimum panel with both 10% and 20% cut-offs in resection specimens from patients with PBA.

## Background

Pancreatic ductal adenocarcinoma (PDAC) is the fifth most common cause of cancer death in the UK with a 5-year survival of only 2% [1]. This poor prognosis is partly due to late clinical presentation with advanced disease, when the treatment options are limited and relatively ineffective [2]. Surgical resection is the only curative option but is only available to 15-20% patients with localised disease [3,4]. The remainder with locally advanced and/or metastatic disease are offered palliative chemotherapy, radiotherapy and/or best supportive management [2,3]. Adenocarcinomas of the head of pancreas and extra-hepatic cholangiocarcinomas (CCC) present similarly most often with jaundice, pain or weight loss [5]. Morphological similarities in addition to generally poor prognosis for both diseases enable PDAC to be grouped with extrahepatic CCC to form so-called pancreatico-biliary adenocarcinomas (PBA).

Diagnosis of PBA relies upon a combination of radiological and cytology or pathology findings [6-10]. Confirmatory tissue diagnosis is necessary before chemotherapy or radiotherapy treatment, however a biopsy specimen is not always required for resection when the suspicion of cancer is high; as generally, the resection will provide therapeutic benefit, and substantially delaying surgery to confirm a diagnosis may deny potentially curative treatment [9,11-15].

Endoscopic ultrasound-guided fine needle aspiration (EUS-FNA) is normally used to obtain cytological samples from pancreatic mass lesions, while endoscopic retrograde cholangio-pancreato-graphy (ERCP) biliary brushings are used for cytology collection from strictures of pancreatico-biliary (PB) ducts [16-18]. Cytological analysis requires the distinction of malignant PB epithelial cells from reactive pancreatic and bile duct cells as well as other gastrointestinal contaminants. This task requires tremendous expertise and can be difficult for both quantitative and qualitative reasons [19]. Quantitatively, the cytological sample obtained may be of low cellularity with few, or even no malignant epithelial cells amongst a variety of cell types. Qualitatively, PBA cells can be morphologically similar to reactive PB cells, especially in well-differentiated adenocarcinomas. Chronic reactive changes arising from atrophy or inflammation in pancreatitis or cholangitis are common, and also make diagnosis of adenocarcinoma difficult.

Expressing these issues statistically, the reported sensitivity of EUS-FNA ranges from 78%-95% with specificity reported to be 75-100% [17,18,20-25]. Though the specificity of biliary brush cytology is high, the sensitivity can be low with ranges of 46% to 73% reported [10,16,26,27]. The sensitivity of EUS-FNA cytology decreases to 62% in chronic pancreatitis and to only 50% in cases of chronic pancreatitis with obstructive jaundice [28]. Thus, a tissue diagnosis is not achieved in a significant proportion of PBA cases. Hence, an unmet clinical need exists for the diagnosis of PBA from cytological samples obtained at EUS-FNA and ERCP.

One potential way of improving cytological diagnosis is to use immunohistochemical (IHC) biomarkers as an adjunct to cytology in difficult to diagnose cases. IHC is a technique widely used in diagnostic pathology that enables the observation and localisation of protein expression simultaneously in tissue and cellular compartments [29]. Diagnostic IHC biomarkers have been investigated both as single biomarkers and as part of biomarker panels to improve the diagnosis of PDAC, but to date none has entered into routine clinical practice [30-37]. We performed a meta-analysis of potential PDAC IHC diagnostic biomarkers [38] aiming to generate a list of biomarkers assessed in either surgical or cytology specimens, where PDAC was compared with normal pancreas and/or chronic pancreatitis. Meta-analytical results showed KOC, S100P, mesothelin and MUC1 to be high-ranking candidates. These biomarkers have not entered into routine clinical practice partly because they were investigated in separate studies with relatively small sample sizes and without uniform and clinically appropriate thresholds for positivity.

We sought to investigate the utility of these four candidate biomarkers in the characterisation of PBA, including both PDAC and CCC. CCC has been included because it often enters the clinical and pathological differential diagnosis; and its positive biomarkers are generally shared with PDAC [39-42]. The aim was to identify a clinically useful diagnostic biomarker or panel of biomarkers with a robust cut-off for positivity that could potentially be taken forward for validation in PBA cytology samples.

A biomarker panel of KOC, S100P and mesothelin with at least 2 biomarkers positive was found to be an optimum panel with both 10% and 20% cut-off achieving almost 100% sensitivity and specificity in resection specimens from patients with PBA.

## Methods

### Tissue Microarrays

Histological sections from tissue microarrays (TMAs) containing samples from 99 surgically resected PBA patients (PDAC = 85, CCC = 14) were used for IHC. All resectional surgery was performed in the West of Scotland Pancreatic Unit, Glasgow Royal Infirmary, UK, during a 10-year period (1st June 1995 to 31st July 2004). Formalin fixed paraffin embedded (FFPE) tumour specimens were archived in the Department of Pathology, Glasgow Royal Infirmary and were used for the construction of TMAs. The construction and use of these TMAs has been previously described [43]. Ethical approval has been granted by the North Glasgow University Hospitals NHS Trust Ethics Committee and by the National Health Service Greater Glasgow and Clyde Ethics Committee. This ethics approval includes the use of archival pathology specimens, where the patients were not given the opportunity to donate their tissue. These TMAs contain five tissue cores (3 tumours and 2 normal) for each patient. Tumour cores are adenocarcinoma cores from PBA patients, whereas normal cores are from adjacent normal pancreatic ducts and acini.

### Immunohistochemistry

IHC was performed for KOC, S100P, mesothelin and MUC1 on our TMA cohort, on an automated platform. Details of the antibodies, antibody concentrations and IHC conditions are shown in Table 1.

**Table 1:** Details of the immunohistochemistry methodology for four antibodies

**Antibody**	**Company**	**Clone of antibody**	**Host animal**	**Antigen retrieval**	**Antibody dilution**	**Incubation temperature**	**Duration of incubation**
**KOC/IMP3**	DAKO	L523S, 69.1	Mouse Monoclonal	HIER* (Citrate buffer PH 6)	1:50	25°C	60 min
**S100P**	BD Biosciences	16	Mouse monoclonal	Proteinase K (10 minutes)	1:100	25°C	60 min
**Mesothelin**	Novocastra	5B2	Mouse monoclonal	HIER (Citrate buffer PH 6)	1:20	25°C	60 min
**MUC1**	Novocastra	MA695	Mouse monoclonal	HIER (Citrate buffer PH 6)	1:200	25°C	60 min

*HIER= Heat Induced epitope retrieval.

### Scoring of tissue specimens

Stained TMA sections were scanned (Hamamatsu Slide Scanner) and images uploaded in Distiller 2.2 (Leica Biosystems). Microscopic analysis was undertaken blinded to diagnosis or other parameters. IHC staining of all cores was assessed by one author (AA); a second author (KAO) double-scored approximately 15% of cores, in a blinded fashion, as audit. All scores were exported in an Excel spreadsheet from Distiller 2.2 for analysis. A semi-quantitative Histoscore [0 ×% negative cells + 1 ×% weakly stained cells + 2 ×% moderately stained cells + 3 ×% strongly stained cells] was generated for statistical analysis. This Histoscore thus has a range of possible scores between 0 and 300.

### Statistics and data analysis

The mean expression of each biomarker in the PBA tumour cores was compared with the mean expression in normal tissue cores. Statistical significance was calculated using the independent sample t-test to generate the p value. The independent sample t test was used rather than the paired sample t test because a full set of matching tumour and normal tissue cores was not available for approximately 5% of patients. This was due to loss of tissue cores during processing, which is expected in a proportion of samples. Sensitivity/specificity analyses were carried out for biomarkers, both individually and in panels of 2–4 biomarkers, and compared. We used two different panel approaches for sensitivity/specificity analysis. One approach assigns the case into the positive category if the tumour expresses only one biomarker in the panel. The other approach assigns the case into the positive category if the tumour shows staining for at least 2 biomarkers in the panel.

A combined summary receiver operating characteristic (SROC) curve was generated to compare different panels of biomarkers. P value <0.05 was considered statistically significant. SPSS-19 and RevMan-5.1 were used for statistical analysis.

## Results

We first performed IHC for each of the four biomarkers on microarrays of normal and tumour tissue from patients with PBA. To fully assess the clinical usefulness of these biomarkers we wanted to analyse the expression of all four biomarkers in PBA versus normal tissue. Moreover, by combining various biomarkers in panels, we hypothesised that we would be able to determine the combination of biomarkers that would deliver the best diagnostic sensitivity and specificity.

### Staining characteristics of biomarkers

**Figure 1:**
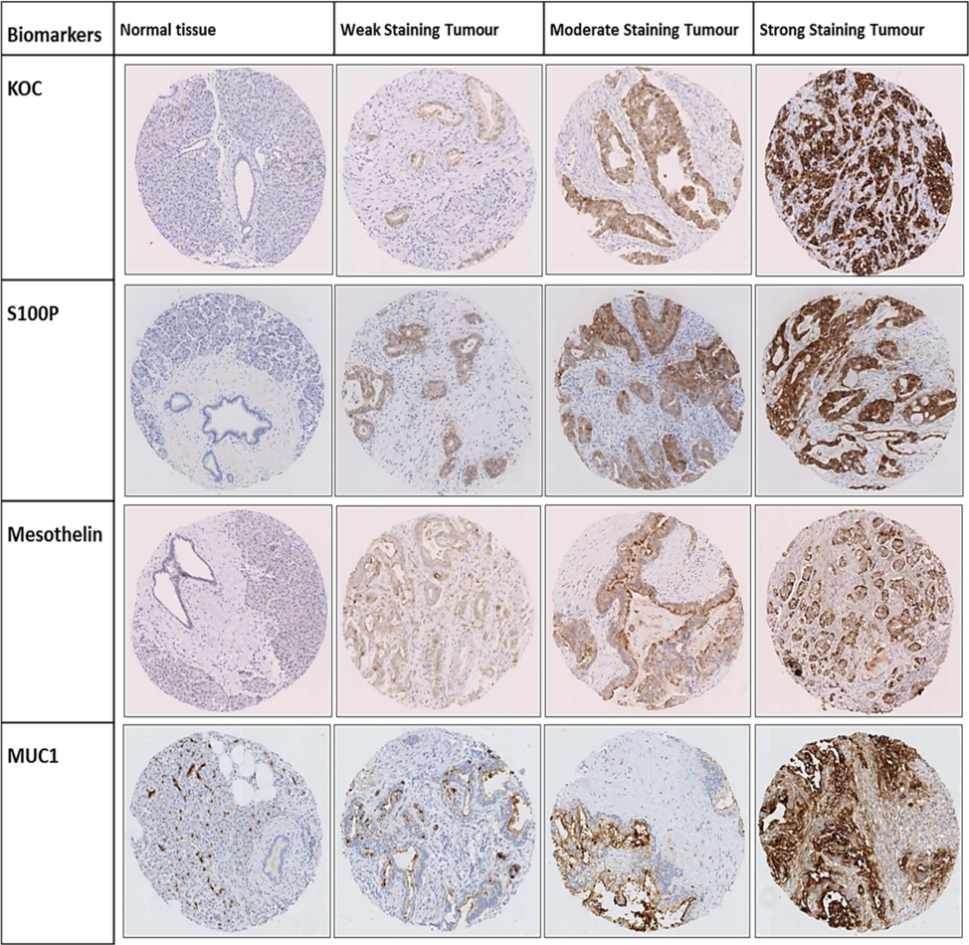
**Representative images of staining of all four biomarkers in normal tissue (normal pancreatic tissue) and range of staining intensities (weak, moderate and strong) in tumour tissue from tissue microarray cores.**

For each marker assessed in the PB TMAs, IHC staining was seen only in epithelial cells. As expected, KOC expression was observed in the cytoplasm; S100P was expressed in the cytoplasm and nucleus, while mesothelin and MUC1 expression was cytoplasmic and membranous (Figure 1). In general, we observed moderate to strong intensity of staining for KOC, mesothelin, S100P and MUC1 in PBA. Moreover, for all four biomarkers we observed significantly higher expression in tumour versus normal tissue (non-neoplastic ducts or pancreatic acinar tissue). The mean percentage positivity for biomarkers in tumour vs. normal tissue was as follows: for KOC 74% vs. 0.4%; for S100P 75% vs. 0.3%; for mesothelin 75% vs. 4%; and for MUC1 75% vs. 18% (Table 2, p < 0.0001 for all tumour vs. normal comparisons). When scored simply as the percentage of positive staining cells per tumour core, we observed similar results for all four biomarkers in tumour tissue. As shown in Table 2, the mean percentage of positive carcinoma cells in tumour tissue was 74% for KOC, 75% for S100P, 73% for mesothelin and 75% for MUC1.

**Table 2:** **Summary statistics of KOC**, **S100P**, **mesothelin and MUC1 expression on a per core basis comparing pancreatico**-**biliary adenocarcinomas with normal tissue**

**Biomarkers**		**Pancreaticobiliary adenocarcinoma**	**Normal tissue**	**P value**
**KOC**				
Positivity*	Mean	74%	0.4%	<0.0001
	Median	100%	0%	
*Histoscore*	Mean	150	0.5	<0.0001
	Median	180	0	
**S100P**				
*Positivity*	Mean	75%	0.3%	<0.0001
	Median	100%	0%	
*Histoscore*	Mean	165	0.3	<0.0001
	Median	180	0	
**Mesothelin**				
*Positivity*	Mean	73%	4%	<0.0001
	Median	90%	0%	
*Histoscore*	Mean	115	4	<0.0001
	Median	110	0	
**MUC1**				
*Positivity*	Mean	75%	18%	<0.0001
	Median	90%	10%	
*Histoscore*	Mean	193	48	<0.0001
	Median	200	30	

**Note**: *Positivity (percentage of positive cells with any staining intensity in tumour and normal tissue); P value (shows the statistical significance of the difference in expression of a biomarker in tumour vs. normal tissue); Positivity range (0–100), Histoscore range (0–300).

By employing a Histoscore scoring method, which takes into account both the extent of expression across the tissue core, and the staining intensity, we were able more to perform a more comprehensive analysis of our biomarkers. Utilizing this method to score the degree and intensity of staining revealed variance of expression of the different biomarkers. As shown in Table 2, the mean tumour tissue versus mean normal tissue Histoscore for MUC1 was 193 vs. 48, while for S100P, KOC and mesothelin, the mean tumour tissue versus mean normal tissue Histoscores were 165 vs. 0.3, 150 vs. 0.5 and 115 vs. 4 respectively.

Although one biomarker, MUC1, was expressed in normal tissue as evidenced by the mean percentage positivity of 16% of normal cells in normal tissues, the expression of the other three biomarkers was very low in normal tissue (Table 2). Furthermore, there were no significant differences in biomarker expression between normal ducts only and normal ducts and acini together (see Additional file 1). Thus, IHC staining using these markers could greatly facilitate interpretation of cytology samples.

Biomarkers expression was also assessed in PDAC compared to CCC as shown in Additional file 2. The expression of all four biomarkers is similar in PDAC and CCC and thus there is no statistically significant difference in the mean expression of biomarkers between these two tumour types (p > 0.05, independent sample t test). Therefore, for sensitivity and specificity analyses PDAC and CCC were grouped as PBA.

### Sensitivity and specificity analysis

#### *Establishing cut-offs from ROC curve analysis*

**Figure 2:**
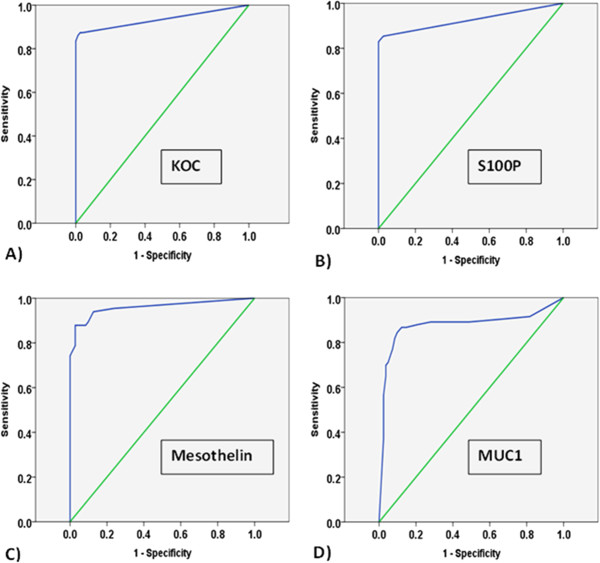
**ROC curves based on percentage of cells positive for any staining (weak, moderate or strong), in tumour and normal cases, for four biomarkers (A) KOC, (B) S100P, C) mesothelin and D) MUC1.**

The sensitivity and specificity of these four biomarkers were evaluated using five cut-offs (thresholds) for positivity as follows: 5% positive cells of any staining intensity; 10% positive cells of any staining intensity; 20% positive cells of any staining intensity; moderate or strong staining of any cells; and Histoscore ≥20. Three of these cut-offs were based on percentage of positive cells and identified by ROC curve analysis. The sensitivity of each biomarker was plotted against 1 – specificity, and ROC curves with coordinates were generated for all four biomarkers (Figure 2). The area under the curve was 0.93 (0.88-0.97, 95% CI) for KOC, 0.92 (0.85-0.99, 95% CI) for S100P, 0.95 (0.92-0.99, 95% CI) for mesothelin, and 0.87 (0.81-0.93, 95% CI) for MUC1. Based on percentage of positive cells in the tumour compared with normal cores, ROC curve analysis allowed us to assess potential cut-offs, from 5% positive cells to 95% positive cells, with their corresponding sensitivity and specificity values for all four biomarkers (Figure 2 and Additional file 3). Three best cut-offs; 5%; 10% or 20% of positive cells of any staining intensity were selected based on their sensitivity and specificity values.

The fourth cut-off was based on moderate to strong staining intensity (+2/+3 staining) in any of the cells. This was selected as moderate to strong staining was expected to be easily interpreted by pathologists. Interestingly, cases with +2/+3 staining for all four biomarkers have more than 20% cells positive for each of the four biomarkers. Indeed patients with +2/+3 staining have only 5 cases with less than 50% of cells positive for MUC1, 2 cases in which KOC was expressed in fewer than 50% of cells, and only 1 case each for mesothelin and S100P staining with less than 50% positivity.

The fifth cut-off was based on a Histoscore value of 20 (HS20), and was selected from ROC curve analysis (see Additional file 4).

#### *Sensitivity and specificity of candidate biomarkers*

**Figure 3:**
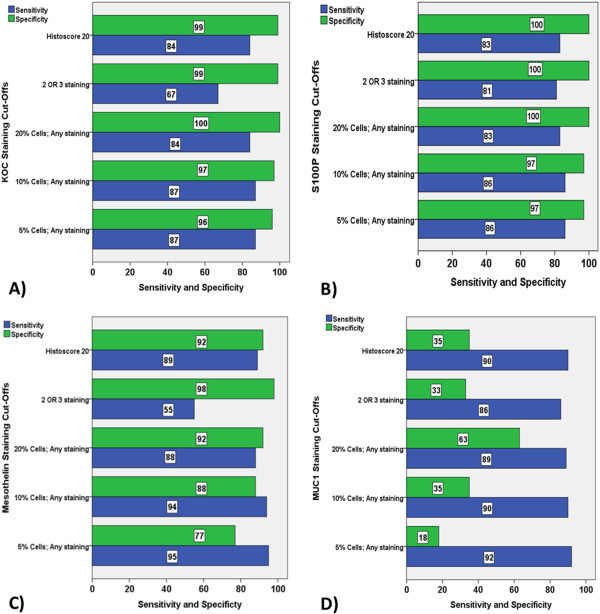
**Sensitivity and specificity analysis of biomarkers for the diagnosis of pancreatico**-**biliary adenocarcinoma compared to normal tissue, based on five cut-offs for positivity: 5% positive cells of any staining intensity; 10% positive cells of any staining intensity; 20% positive cells of any staining intensity; 2 OR 3 intensity i.e. moderate or strong staining of cells; and Histoscore 20.** Analysis is presented for **A)** KOC, **B)** S100P, **C)** mesothelin and **D)** MUC1.

The sensitivities and specificities of all four biomarkers were calculated using these five cut-offs, as shown in Figure 3. KOC expression appears to show reasonably high sensitivity and specificity for all cut-offs except for the cut-off based on +2/+3 staining, which resulted in low sensitivity of only 67%. The 20% positive cells cut-off achieves marginally better sensitivity (84%) and specificity (100%) values compared with other cut-offs for KOC (Figure 3A). S100P appears to have similar sensitivity and specificity values for all five cut-offs with the 20% cut-off again achieving better combination of specificity and sensitivity, with values of 83% sensitivity and 100% specificity (Figure 3B).Applying the five cuts-offs to the analysis of mesothelin expression resulted in significantly different sensitivity and specificity values, however, the best combination was again achieved using the 20% cut-off, with 88% sensitivity and 92% specificity (Figure 3C). Although the sensitivity of MUC1 as biomarker is high across all cut-offs, its specificity is unacceptably low for all cut-offs, with a range of 18%-63% compromising the diagnostic accuracy of MUC1 (Figure 3D).

### Sensitivity and specificity analysis using biomarker panels

We next wanted to assess the sensitivity and specificity achieved using panels of biomarkers. The 10% and 20% cut-offs were selected for this investigation, based on their diagnostic performance.

#### *Analysis based on one positive biomarker in a panel*

**Figure 4:**
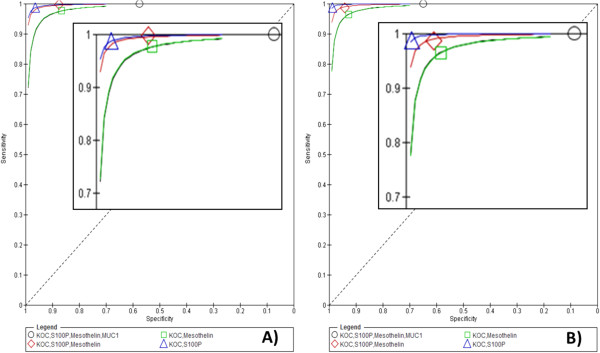
*Combined Summary ROC curves for 10% (A) and 20% (B) cut-offs if only one biomarker was required to be positive in a panel. Four panels of biomarkers were compared. Panel 1 - KOC, S100P, Mesothelin and MUC1; Panel 2 - KOC, S100P, Mesothelin; Panel 3 - KOC, S100P; Panel 4 - KOC, Mesothelin. *Summary ROC curves plot sensitivity against specificity and draw a summary line depicting combined sensitivity and specificity of a panel. Combined Summary ROC curves compare different panels to show the most “accurate” panel. The summary line at the top left corner shows the biomarker which is most accurate compared to others lying lower and further to the right. This enables the most accurate panel to be identified.

We first assessed the sensitivity and specificity achieved when one biomarker in a panel is positive, using four different panels (Table 3). These panels were: a panel comprising all four biomarkers; a panel of three biomarkers (KOC, S100P and mesothelin); and two panels of two biomarkers (KOC and mesothelin, KOC and S100P). As expected, a panel of all four biomarkers achieved very low specificity of 40% and 65% respectively for 10% and 20% cut-offs, due to the low specificity of MUC1 as a biomarker. A panel of KOC, S100P and mesothelin achieved sensitivity/specificity of 100%/88% for the 10% cut-off and 99%/94% for the 20% cut-off. A panel of KOC and mesothelin achieved sensitivity/specificity of 97%/87% and 96%/93% for the 10% cut-off and 20% cut-offs, respectively. Finally, a panel of KOC and S100P achieved sensitivity/specificity of 98%/96% for the 10% cut-off and 99%/99% for the 20% cut-off.These panels were compared by combined SROC curve, using both the 10% cut-off (Figure 4A) and 20% cut-offs (Figure 4B). The combined SROC curve shows that a panel of KOC and S100P is superior to the other panels for both 10% and 20% cut-offs.

**Table 3:** **Panels of biomarkers used for analysis of specificity and sensitivity**, **using 10**% **positive cells and 20**% **positive cells as cut**-**off thresholds for positivity**

**10% positive cells as cut**-**off**		
**Panels**	**Sensitivity**	**Specificity**
**KOC, S100P, Mesothelin, MUC1**	100%	40%
**KOC, S100P, Mesothelin**	100%	88%
**KOC, Mesothelin**	97%	87%
**KOC**, **S100P**	98%	96%
**KOC**, S100P, Mesothelin*	100%	99%
**20% positive cells as cut-off**		
**Panels**	**Sensitivity**	**Specificity**
**KOC, S100P, Mesothelin, MUC1**	100%	65%
**KOC, S100P, Mesothelin**	99%	94%
**KOC, Mesothelin**	96%	93%
**KOC, S100P**	99%	99%
KOC, S100P, Mesothelin*	100%	100%

**Note**: *At least 2 biomarkers required to be positive in this panel. In the rest of the panels only one biomarker was required to be positive in a panel.

#### *Analysis based on two or more positive biomarkers in a panel*

Finally, one biomarker panel comprising KOC, S100P and mesothelin was tested for sensitivity and specificity when at least 2 biomarkers in the panel are positive. This panel achieved almost 100% sensitivity/specificity for both 10% and 20% cut-offs (Table 3). Taken together, our results show that this panel could be used to improve diagnosis of PBA in difficult to diagnose cases.

## Discussion

Four potentially diagnostic biomarkers, KOC, S100P, mesothelin and MUC1, were investigated in a relatively large cohort of PB patients (n = 99). The expression levels of KOC, S100P and mesothelin were high in tumour tissue compared with normal tissue. The diagnostic accuracy (sensitivity and specificity) of KOC and S100P individually was greater than that of mesothelin and MUC1. A panel of KOC, S100P and mesothelin with at least 2 biomarkers positive achieved almost perfect diagnostic accuracy in the differentiation of carcinoma from normal tissue.

IHC biomarkers have previously been investigated in surgical and cytological cohorts but none is yet routinely used for improving the diagnosis of PBA [35-37,41,44-46]. There are six significant reasons delaying the clinical translation of diagnostic biomarkers in PBA and other cancers. These reasons and our approach to address them are outlined below.

Firstly, a plethora of research exists on diagnostic IHC biomarkers coming from the bench assessed in pilot studies. There are many excellent papers but fewer validation studies for biomarkers that have shown promising results. Clearly, validation is important for future clinical application. Therefore, we performed a meta-analysis on diagnostic IHC biomarkers for PDAC [38], to review, quantify and assess the performance of already existing biomarkers and to try and identify superior candidate biomarkers.

The biomarkers derived from the meta-analysis in PDAC were applied in our study to both PDAC and CCC samples. Separate meta-analysis was not performed for CCC, because there are relatively few published papers on biomarkers in CCC (approximately 20-fold fewer than for PDAC; PubMed search in June 2014, unpublished data). However, those papers which are available for CCC suggest that the biomarker expression profile is similar to PDAC. To our knowledge, all of the known positive biomarkers for PDAC (versus corresponding normal tissue), including MUC1, P53, CK17, mesothelin, fascin, MUC4, 14-3-3σ and prostate stem cell antigen, show similar IHC expression in CCC (versus corresponding normal tissue) [39-42].

For these reasons, we focused on PDAC for the identification of potential diagnostic biomarkers then tested the resulting candidates in TMAs containing tissue from both PDAC and CCC using IHC. From our meta-analysis, we selected KOC [36,37,44,47], S100P [32,35,48], mesothelin [30,49,50] and MUC1 [31,40,51] for investigation.

We found that expression of these biomarkers was similar in PDAC and CCC (Additional File 2): our results therefore supporting the previous literature [39-42].

Second, the sample size for studies investigating diagnostic biomarkers for PDAC is relatively small (median sample size, n = 48 from 57 articles). Moreover, matched normal tissue for most of the carcinoma case is not always available, leading to even smaller sample sizes for calculating biomarker specificity. Therefore, statistical power is relatively low and subsequently potentially useful biomarkers may be ignored. Our relatively larger sample size of 99 PBA cases (n = 99 adenocarcinomas and n = 99 matched normal tissue for each case; total n = 198) provided a solid platform for investigating these diagnostic IHC biomarkers.

Third, the lack of a standardised scoring system and absence of a uniform cut-off (threshold) for the interpretation of IHC remains problematic. Thus, researchers use a variety of traditional and novel scoring systems and diverse cut-offs, making the adoption of scoring systems and cut-offs potentially challenging for the pathologists [30,35,37,41,46,49,52-54]. We systematically chose cut-offs from ROC curve analysis to fully explore the diagnostic potential of all four biomarkers. These cut-offs provide an opportunity for the pathologists to select the best threshold that is more clinically applicable and has the potential to be routinely used in pathology. Three of these cut-offs are based on proportion of positive cells (5%, 10% and 20%) with staining of any intensity. The fourth cut-off is based on any proportion of cells exhibiting moderate and strong staining intensity, and the fifth cut-off is based on a Histoscore of 20. Notably, the 20% cut-off and Histoscore 20 provide reasonable sensitivity and specificity values for PBA diagnosis. A higher Histoscore value could potentially lead to more false negatives in tumour cases, therefore, a low cut-off value of 20 was chosen. Clearly, this cut-off will remove the probability of false negative and should increase the diagnostic confidence of pathologists for higher Histoscore values. For example, a Histoscore value of 200 for a biomarker in a suspicious case might help the pathologist to diagnose a tumour with confidence and with a much higher specificity.

Fourth, most of the IHC diagnostic biomarkers have been investigated individually [32,46,47,55,56], with few studies reporting the utility of biomarker panels [30,36]. We carefully selected candidate biomarkers reported in different studies (KOC, mesothelin, S100P and MUC1) for investigation in a single experimental setting. Investigation of these biomarkers in a single cohort gave us the opportunity to compare biomarkers, and then further explore their diagnostic accuracy in a panel. Expectation from an ideal diagnostic biomarker is its ability to identify the diseased population (sensitivity) and exclude the normal population (specificity) in 100% cases. However, no single biomarker is 100% perfect; therefore these biomarkers were investigated in various combinations, to select an optimum panel for potential clinical application. For example, the individual sensitivity/specificity of KOC and S100P at a cut-off of 20% positive cells was 84%/100% and 83%/100% respectively. However, using a panel of KOC and S100P improved sensitivity to 99% without compromising the specificity (99%).

Furthermore, using a panel of KOC, S100P and mesothelin with at least 2 positive biomarkers achieved almost 100% sensitivity and specificity for both 10% and 20% cut-offs. This approach would assign a patient into the tumour positive category if 2 or more biomarkers are positive, possibly giving more assurance to the pathologist before assigning patient into positive category. Moreover, a combination of KOC, S100P and mesothelin antibodies should stain all major cellular compartments (cell membrane, nucleus and cytoplasm). Clinically, a cytology sample comprises a mixed population of cells and this panel will stain malignant cells more intensely making the interpretation of IHC convenient for the pathologist. The possible additional advantage of KOC is that it is not expressed in the contaminating gastrointestinal epithelial cells that are usually present in cytological samples [44,57]. Our data also confirm the lack of expression of KOC in normal duodenum. Taken together, our results reinforce the reported sensitivity/specificity values for KOC, S100P and mesothelin [30,35,37,57] and further explores their utility as a panel.

The fifth reason is that different research groups use different IHC experimental conditions, primary antibodies, clones, dilutions and manual/automated platforms that could potentially lead to a diverse range of sensitivity and specificity values for biomarkers [30,45,54,58-60]. We thoroughly searched the literature for IHC parameters for KOC, S100P, mesothelin and MUC1. Those IHC parameters that achieved superior diagnostic accuracy were selected and further optimised in our histology laboratory before staining our cohort.

Finally, an important requirement for biomarker translation to the clinic is independent validation with the aim of improving already existing diagnosis. Purposeful validation in surgical and cytological tissue from PBA cohorts and subsequent prospective clinical study on cytological samples is deficient. Therefore, as an important step for potential clinical translation we investigated KOC, S100P, mesothelin and MUC1 in a surgical cohort of PBA patients with promising results for KOC, S100P and mesothelin as a biomarker panel.

The next step forward is to possibly investigate these biomarkers in a retrospective and then in a prospective cohort of cytology samples. This manuscript systematically attempted to answer all six major reasons hindering the clinical translation of diagnostic IHC biomarkers for pancreatic cancer. It also provides future direction and work packages to be performed before these diagnostic biomarkers can be used in day-to-day pathology practice.

## Conclusions

Our results demonstrate that a biomarker panel of KOC, S100P and mesothelin is capable of categorising PB malignancy with high diagnostic accuracy in resection specimens. We plan to investigate this panel in archival cytological samples. As an adjunct to cytology, this panel has the potential to augment the categorisation for challenging diagnostic cases in routine clinical practice.To our knowledge, this is the first study of PB literature that identified cut-offs systematically for diagnostic purposes and used stringent panels to identify an optimum biomarker panel.

## Abbreviations

PDAC: Pancreatic ductal adenocarcinoma; PBA: Pancreatico-biliary adenocarcinomas; ERCP: Endoscopic retrograde cholangio-pancreato-graphy; EUS-FNA: Endoscopic ultrasound-guided fine needle aspiration; IHC: immunohistochemistry; ROC curve: receiver operating characteristic curve; TMA: tissue microarrays; SROC: summary receiver operating characteristic.

## Competing interests

The authors declare that they have no competing interests.

## Authors’ contributions

KAO, AA, FRD and VB participated in the conception and study design. AA, VB, SD carried out data collection. AA, VB and CN carried out immunostaining. AA, NBJ, JPM, JSG and KAO contributed in data analysis and interpretation. NBJ, JPM, OJS, CRC, CJM, FRD, JSG, AA, KAO were involved in manuscript preparation and provided their critical comments from surgical, pathological and scientific perspectives. All authors have read and approved the final manuscript.

## Pre-publication history

The pre-publication history for this paper can be accessed here:

http://www.biomedcentral.com/1472-6890/14/35/prepub

## Supplementary Material

Additional file 1 Summary statistics of KOC, S100P, mesothelin and MUC1 expression on a per core basis comparing pancreatico-biliary adenocarcinomas with normal ducts and normal ducts & acini together. Click here for file

Additional file 2 Summary statistics of KOC, S100P, mesothelin and MUC1 expression on a per core basis comparing pancreatic ductal adenocarcinoma with cholangiocarcinoma. Click here for file

Additional file 3 Cut-offs resulting from ROC curve analysis based on the percentage of positive cells of any staining intensity (weak, moderate or strong) in tumour and normal cases for four biomarkers KOC, S100P, mesothelin and MUC1. Click here for file

Additional file 4 ROC curves based on histoscores, in tumour and normal cases, for four biomarkers A) KOC, B) S100P, C) mesothelin and D) MUC1. Click here for file
