# Supplementary material for: Expression of KOC, S100P, mesothelin and MUC1 in pancreatico-biliary adenocarcinomas: development and utility of a potential diagnostic immunohistochemistry panel
Source: BMC Clin Pathol. 2014 Jul 23;14:35. doi: 10.1186/1472-6890-14-35 (PMC4112611; doi:10.1186/1472-6890-14-35)
Supplement: Additional file 1 — Summary statistics of KOC, S100P, mesothelin and MUC1 expression on a per core basis comparing pancreatico-biliary adenocarcinomas with normal ducts and normal ducts & acini together. [file 1472-6890-14-35-S1.pdf]

Additional file 1: Summary statistics of KOC, S100P, mesothelin and MUC1 expression on a per core basis comparing pancreatico-biliary adenocarcinomas with normal ducts and normal ducts & acini together

Biomarkers		Pancreatico-biliary Adenocarcinoma	Normal ducts	Normal ducts & acini	P value
**KOC**					
*Positivity**	Mean	74%	0.2%	0.4%	<0.0001
	Median	100%	0%	0%	
Histoscore	Mean	150	0.4	0.5	<0.0001
	Median	180	0	0	
**S100P**					
Positivity	Mean	75%	0.3%	0.3%	<0.0001
	Median	100	0%	0%	
Histoscore	Mean	165	0.3	0.3	<0.0001
	Median	180	0	0	
**Mesothelin**					
Positivity	Mean	73	5%	4%	<0.0001
	Median	90	0%	0%	
Histoscore	Mean	115	5	4	<0.0001
	Median	110	0	0	
**MUC1**					
Positivity	Mean	75	16%	18%	<0.0001
	Median	90	5%	10%	
Histoscore	Mean	193	37	48	<0.0001
	Median	200	14	30	

Note: *Positivity (percentage of positive cells of any staining intensity in tumour and normal tissue); P value (Shows the statistical significance of the difference in expression of a biomarker in tumour vs. normal tissue); Positivity range (0-100), Histoscore range (0-300).
