# Supplementary material for: Expression of KOC, S100P, mesothelin and MUC1 in pancreatico-biliary adenocarcinomas: development and utility of a potential diagnostic immunohistochemistry panel
Source: BMC Clin Pathol. 2014 Jul 23;14:35. doi: 10.1186/1472-6890-14-35 (PMC4112611; doi:10.1186/1472-6890-14-35)
Supplement: Additional file 2 — Summary statistics of KOC, S100P, mesothelin and MUC1 expression on a per core basis comparing pancreatic ductal adenocarcinoma with cholangiocarcinoma. [file 1472-6890-14-35-S2.pdf]

Additional file 2: Summary statistics of KOC, S100P, mesothelin and MUC1 expression on a per core basis comparing pancreatic ductal adenocarcinoma with cholangiocarcinoma

Biomarkers		Pancreatic ductal adenocarcinoma	Cholangiocarcinoma	P value
**KOC**				
*Positivity**	Mean	74%	60%	0.09
	Median	100%	90%	
Histoscore	Mean	155	131	0.33
	Median	180	120	
**S100P**				
Positivity	Mean	75%	80%	0.38
	Median	100%	100%	
Histoscore	Mean	160	180	0.15
	Median	150	190	
**Mesothelin**				
Positivity	Mean	72%	75%	0.65
	Median	90%	90%	
Histoscore	Mean	120	135	0.37
	Median	105	140	
**MUC1**				
Positivity	Mean	76%	72%	0.58
	Median	90%	100%	
Histoscore	Mean	195	198	0.91
	Median	220	260	

Note: *Positivity (percentage of positive cells with any staining intensity in tumour tissue); P value, independent sample t test (shows the statistical significance of the difference in expression of these biomarkers in pancreatic ductal adenocarcinoma vs. cholangiocarcinoma); Positivity range (0-100), Histoscore range (0-300).
