# Supplementary material for: Expression of KOC, S100P, mesothelin and MUC1 in pancreatico-biliary adenocarcinomas: development and utility of a potential diagnostic immunohistochemistry panel
Source: BMC Clin Pathol. 2014 Jul 23;14:35. doi: 10.1186/1472-6890-14-35 (PMC4112611; doi:10.1186/1472-6890-14-35)
Supplement: Additional file 3 — Cut-offs resulting from ROC curve analysis based on the percentage of positive cells of any staining intensity (weak, moderate or strong) in tumour and normal cases for four biomarkers KOC, S100P, mesothelin and MUC1. [file 1472-6890-14-35-S3.pdf]

Additional file 3: Cut-offs resulting from ROC curve analysis based on the percentage of positive cells of any staining intensity (weak, moderate or strong) in tumour and normal cases for four biomarkers KOC, S100P, mesothelin and MUC1.

**Cut-offs***	**KOC**		**S100P**		**Mesothelin**		**MUC1**	
	**Sensitivity**	**Specificity**	**Sensitivity**	**Specificity**	**Sensitivity**	**Specificity**	**Sensitivity**	**Specificity**
**5%**	87	96	85	100	95	76	92	18
**10%**	87	98	86	97	94	87	90	34
**20%**	84	100	84	100	88	92	89	62
**35%**	81	100	80	100	82	97	87	85
**45%**	77	100	80	100	79	97	84	90
**55%**	72	100	78	100	73	100	77	93
**65%**	71	100	71	100	70	100	71	95
**70%**	70	100	66	100	67	100	70	96
**80%**	68	100	59	100	56	100	65	96
**90%**	63	100	56	100	53	100	53	98
**95%**	56	100	54	100	42	100	40	98

***** These cut-offs are based on percentage positivity (percentage of positive cells of any staining intensity in tumour and normal tissues)
