# Supplementary material for: Expression of KOC, S100P, mesothelin and MUC1 in pancreatico-biliary adenocarcinomas: development and utility of a potential diagnostic immunohistochemistry panel
Source: BMC Clin Pathol. 2014 Jul 23;14:35. doi: 10.1186/1472-6890-14-35 (PMC4112611; doi:10.1186/1472-6890-14-35)

Additional file 4: ROC curves based on histoscores, in tumour and normal cases, for four biomarkers A) KOC, B) S100P, C) mesothelin and D) MUC1.

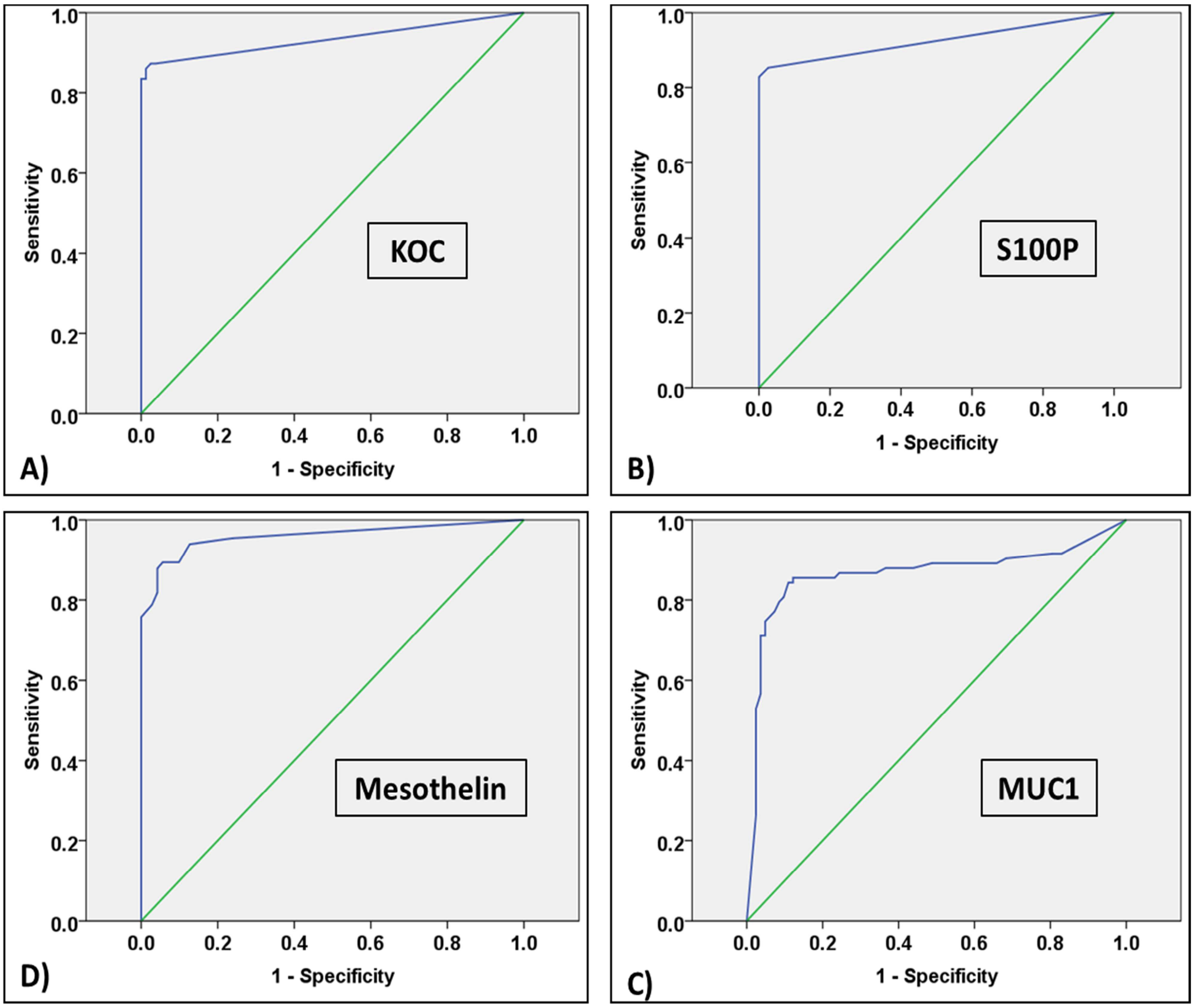

Supplement: Additional file 4 — ROC curves based on histoscores, in tumour and normal cases, for four biomarkers A) KOC, B) S100P, C) mesothelin and D) MUC1. [file 1472-6890-14-35-S4.pdf]
